# Depression Speech Recognition With a Three-Dimensional Convolutional Network

**DOI:** 10.3389/fnhum.2021.713823

**Published:** 2021-09-30

**Authors:** Hongbo Wang, Yu Liu, Xiaoxiao Zhen, Xuyan Tu

**Affiliations:** School of Computer and Communication Engineering, Beijing Key Lab of Knowledge Engineering for Materials Science, University of Science and Technology Beijing, Beijing, China

**Keywords:** depression detection, speech emotion recognition, multi-channel convolution, attention mechanism, deep learning

## Abstract

Depression has become one of the main afflictions that threaten people's mental health. However, the current traditional diagnosis methods have certain limitations, so it is necessary to find a method of objective evaluation of depression based on intelligent technology to assist in the early diagnosis and treatment of patients. Because the abnormal speech features of patients with depression are related to their mental state to some extent, it is valuable to use speech acoustic features as objective indicators for the diagnosis of depression. In order to solve the problem of the complexity of speech in depression and the limited performance of traditional feature extraction methods for speech signals, this article suggests a Three-Dimensional Convolutional filter bank with Highway Networks and Bidirectional GRU (Gated Recurrent Unit) with an Attention mechanism (in short 3D-CBHGA), which includes two key strategies. (1) The three-dimensional feature extraction of the speech signal can timely realize the expression ability of those depression signals. (2) Based on the attention mechanism in the GRU network, the frame-level vector is weighted to get the hidden emotion vector by self-learning. Experiments show that the proposed 3D-CBHGA can well establish mapping from speech signals to depression-related features and improve the accuracy of depression detection in speech signals.

## 1. Introduction

Recently, mental pressure or depression from work and life has become one of the main threats to our health (Kermc et al., 2019). According to relevant statistical results (World Health Organization, 2020), it is estimated that there are more than 350 million patients with depression worldwide, and there are more than 95 million patients with depression in China, basically denoting about 30% of the global average level. However, depression has been plagued by a low recognition rate, low consultation rate, and low treatment rate, and it is highly likely to be seriously underestimated (Huang et al., 2019).

At present, the diagnosis of depression is mainly based on questionnaire surveys, supplemented by doctors' judgment. Its accuracy depends heavily on patient cooperation and physician expertise, and early diagnosis and reassessment of depression are limited. If computer-aided tools can be used to diagnose depression quickly and effectively, with relative safety and without much privacy, it will greatly reduce the difficulty of clinical screening for depression. Therefore, the use of speech information, which is non-invasive and easily accessible, for initial judgment provides a new way for the early screening of depression and to reduce the cost of depression detection to some extent.

Patients with depression are characterized by slow speaking speed, low intonation, weak voice intensity (Kraepelin, 1921), reduced changes in speech features (Cannizzaro et al., 2004), and more pauses (Mundt et al., 2012). At the same time, the changes of voice bandwidth, amplitude, energy and other changes in patients with depression were reduced (Kuny and Stassen, 1993; Mundt et al., 2012), and the spectral characteristics of the patients were also related to the degree of depression (Tolkmitt et al., 1982). Therefore, the feature extraction of speech and the capture of acoustic features will help to better understand depression, because these features are relatively objective and are not deliberately masked easily by individuals. In addition, in the speech signals of depressed patients, strong emotions are not common, such as happiness and anger, but depression, sadness, and calm emotions are extremely common. Therefore, it is of great significance to extract his/her emotional signals from the speaker for the study of depression.

In medical practice, a speech emotion recognition system plays an important role in judging the change of mental state and emotion (Wang et al., 2020). When a patient experiences mood swings or is traumatized, the system will quickly respond and analyze his/her current psychological state. Nantong Tumor hospital (Xu et al., 2018) designed a judgment of psychological feelings of tumor patients based on speech analysis. Different nursing interventions were carried out according to the psychological characteristics of different patients, which promoted their physical and mental health recovery. France et al. proposed that the acoustic characteristics of speech could be used as an indicator of depression and suicide risk (France et al., 2000), and speech could be used to track the emotional changes of depressed patients, so as to serve as the basis for disease diagnosis and treatment.

Since the 1980s, the real research of speech emotion recognition has begun to appear. Bezooijen (Bezooijen et al., 1983) and Tolkmitt (Tolkmitt and Scherer, 1986) initiated the use of acoustic statistical features in the identification of emotions. In 1999, Moriyama proposed the association model of speech and emotion, and applied it to the e-commerce system (Moriyama and Ozawa, 1999), which can collect the user's speech and recognize emotion images. In early studies, research on speech emotions and depression detection mainly included GMMs (Gaussian Mixtures Models) (Yun and Yoo, 2012; Williamson et al., 2013), HMMs (Hidden Markov Models) (Le and Mower Provost, 2013), SVM (Support Vector Machine) (Kao and Lee, 2006; Valstar et al., 2016), and RF (Random Forest) (Svetnik et al., 2003). The process of using the above method to detect depression is to extract features and then use machine learning to study the relationship between features and depression degree. However, in the traditional machine learning method, the selection of features is directly related to the accuracy of depression recognition results. The advantage is that the model can be trained without the need for large amounts of data. The disadvantage is that it is difficult to judge the quality of features, and some key features may be lost, thus reducing the accuracy of identification. In addition, with the emergence of big data in various application fields, from the perspective of timely response, the above solutions have encountered bottlenecks to varying degrees.

With the increase of computing speed, deep learning has become a research hotspot. Compared with traditional machine learning methods, deep learning technology has the advantage of extracting high-level semantic features. Meyer et al. proposed Deep Neural Networks (DNNs) for speech emotion recognition (Stuhlsatz et al., 2011). Han et al. proposed a speech emotion classification system based on a DNN-ELM (Extreme Learning Machine) (Han et al., 2014). The traditional acoustic features of speech were input into the DNN, and the probability distribution of segmental emotional states was generated, from which utterance-level features were constructed, and then ELM was used for classification. Despite the DNN's great success in speech recognition, it still uses personalized features as input, which can be influenced by a variety of speech styles, content of speech, and context, hindering its application in real-world environments that have nothing to do with the speaker. Therefore, it is of great significance to reduce the numerical differences of these personalized features. Bertero et al. applied the Convolutional Neural Network (CNN) (Bertero and Fung, 2017), which plays a great role in the image field, to speech emotion recognition and achieved good results. However, the CNN used is a relatively simple shallow model, and it fails to combine the advantages of the CNN with the temporal correlation characteristics of speech. Due to the Recurrent Neural Network (RNN)'s strong analytical ability on timing problems, Park et al. applied it to speech emotion recognition (Park et al., 2002). Then, researchers improved the RNN and proposed the LSTM (Long Short-Term Memory), GRU (Cho et al., 2014), QRNN (Bradbury et al., 2016), etc. However, one of the major disadvantages of the RNN is that it is difficult to train, and it is easy to cause overfitting problems for small scale emotional data sets. At the same time, some variants try to combine the CNN and RNN into a CRNN (Convolutional Recurrent Neural Network) (Basu et al., 2017) model for speech emotion recognition. The low-dimensional features of speech are taken as the basic features of speech emotion feature extraction. The CNN is used to map the features, and then the LSTM is used to extract sentence-level timing information. Ma et al. proposed a model (Ma et al., 2016) combining the CNN and LSTM to encode depression-related features in the vocal channel to provide a more comprehensive audio representation, and introduced a random sampling strategy to mitigate the bias caused by uneven sample distribution. However, the above two models also only extract low-dimensional features and do not take into account the influence of personalized features. Chao et al. proposed a multimodal depression prediction model based on audiovisual input (Chao et al., 2015). The features of audio and video are extracted and fused into the signs of abnormal behavior, and then the LSTM-RNN is used to describe the dynamic time information. Multi-tasking learning was also used to improve the accuracy of the results.

How to make full use of the original speech signal and improve the accuracy of depression detection is an urgent problem to be solved. For daily conversation speech, the traditional feature extraction method cannot fully express the information. In this paper, a 3D-CBHGA model was proposed and applied to the research of depression. To be clear, our work is designed to enhance existing clinical approaches and provide ancillary support for the diagnosis of depression, rather than issuing a formal diagnosis.

The remainder of this article is organized as follows. In section 2, the process of depression detection with deep learning is described. Section 3 proposes the improved model 3D-CBHGA based on the attention mechanism. The relevant test experiments and discussions are presented in section 4. Finally, the conclusion is given in section 5.

## 2. Problem Description

Speech is a kind of non-invasive, easily accessible information in the clinic (Li and Fu, 2019). The capture of acoustic features that are relatively objective and not easily concealed by individuals will help to better understand depression.

**Figure 1:**
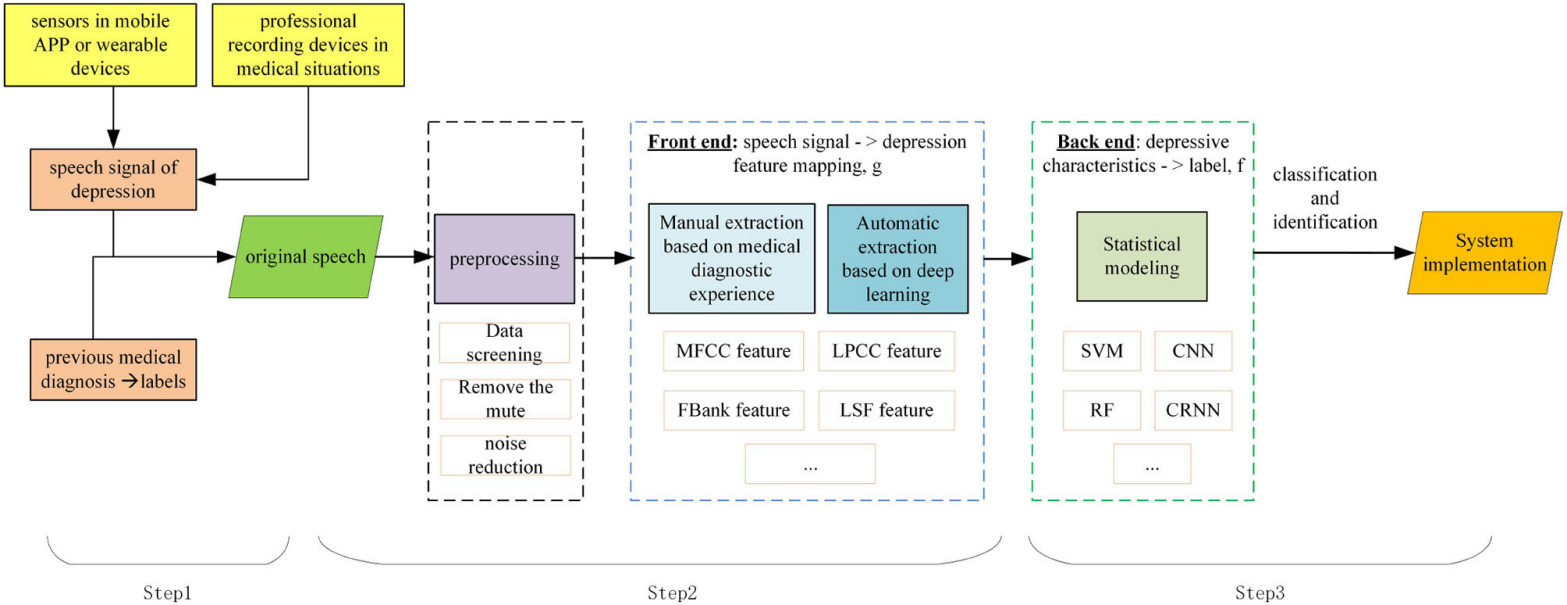
Depression detection based on speech signals.

In the medical analysis of depression, data collection of suspected patients should be carried out first, including standardized data such as pathological electroencephalogram (EEG) signals and questionnaires, as well as irregular data such as expressions, behaviors, and voice intonation. The data should then collated to determine whether patients have depression, or the degree of depression. Similar to the medical situation, depression detection by speech signals is mainly divided into three steps, as shown in Figure 1.

Step 1: Collect the data set. Speech signals can be collected through sensors in mobile APP or wearable devices, or by professional recording devices in medical situations. Firstly, the suspected patient's voice or answers are recorded as a speech signal of depression. The labels of a speech signal depends on its previous medical diagnosis. Depression detection can be considered as a classification or regression problem, depending on how the speech is labeled.

Step 2: Clean and extract speech features associated with depression. Compared with non-depressed patients, depressed patients speak slowly, with low intonation and weak voice intensity. Features can be extracted by combining medical experience and deep learning technology to ensure accuracy and comprehensiveness.

Step 3: Evaluate extracted features. Depending on the type of label, the classifier or regression model can be trained. Thus, the mapping relationship between speech signals and depression is formed, and the depression information can be automatically evaluated.

A speech signal sample is represented as shown in Equation (1).


(1) $$\begin{array}{l} \left[signal_{i},label_{i}\right],i\in N \end{array}$$

Where *signal*_*i*_ represents the *i*-th speech signal, *label*_*i*_ represents its category, and *N* represents the total number of samples.

The speech-based depression detection is represented as shown in Equation (2). Through the study of mapping relations *f* and *g*, the optimal mapping is found, so that samples and labels correspond to each other.


(2) $$\begin{array}{l} f\left(g\left(signal_{i}\right)\right)\to label_{i} \end{array}$$

Where *g* represents the mapping between the input speech *signal*_*i*_ and depression-related features, and *f* represents the mapping between the features and corresponding *label*_*i*_.

**Figure 2:**
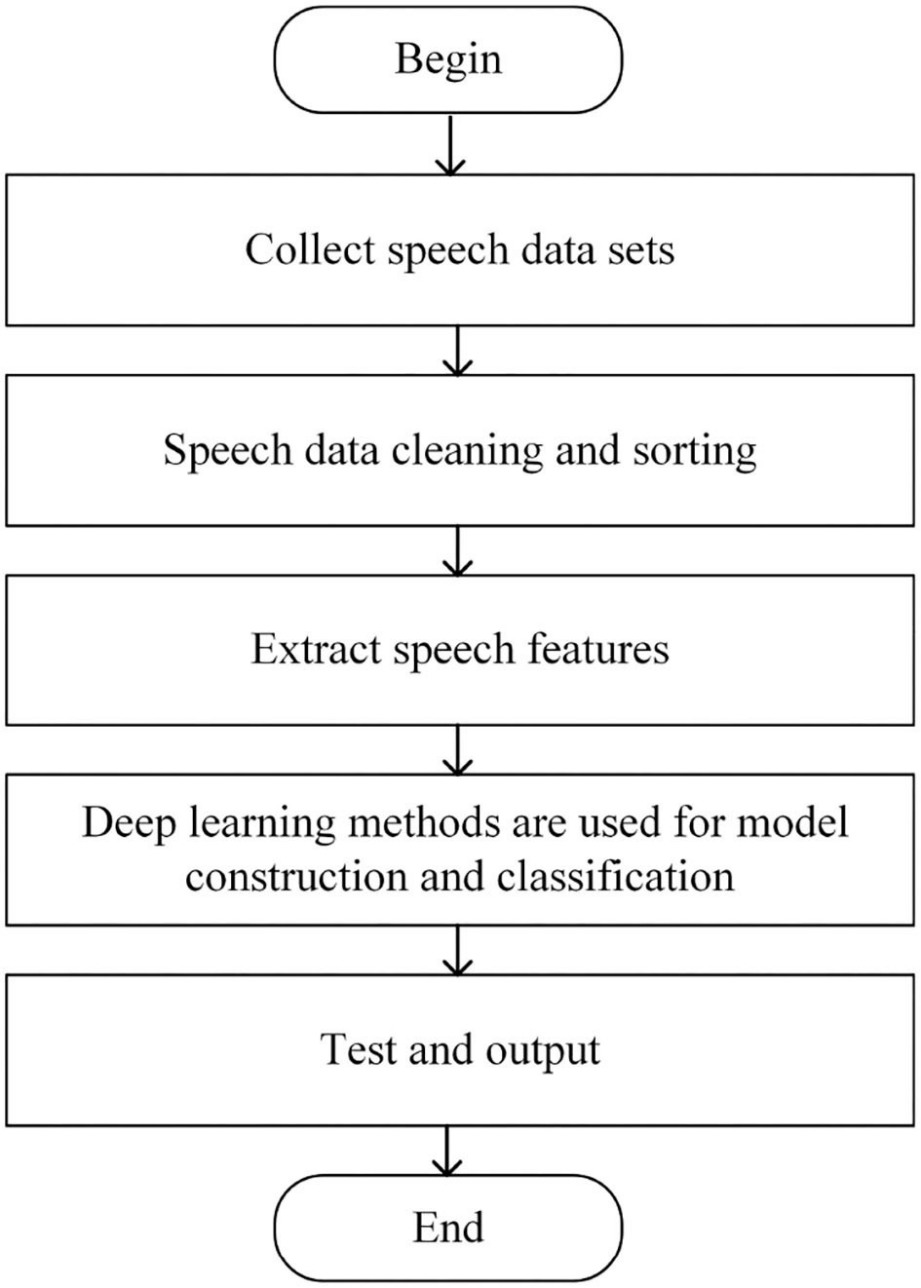
Flow chart of depression detection.

Through the analysis of the depression detection, it can be seen that the key lies in mapping speech signals to depression features. Traditional artificial feature extraction is often unable to find feature sets comprehensively and accurately, which has great limitations. Since deep learning can extract deeper features and shows excellent performance in speech tasks, it is applied to depression detection, and the flow chart is shown in Figure 2.

## 3. Proposed Method

**Figure 3:**
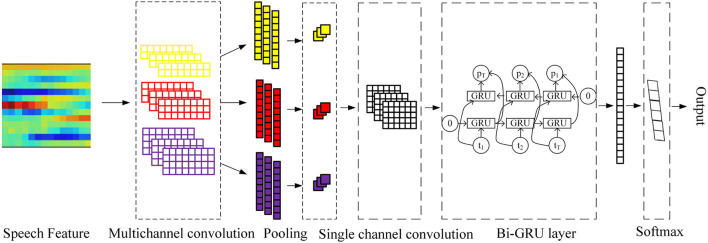
1D-CBBG model.

**Figure 4:**
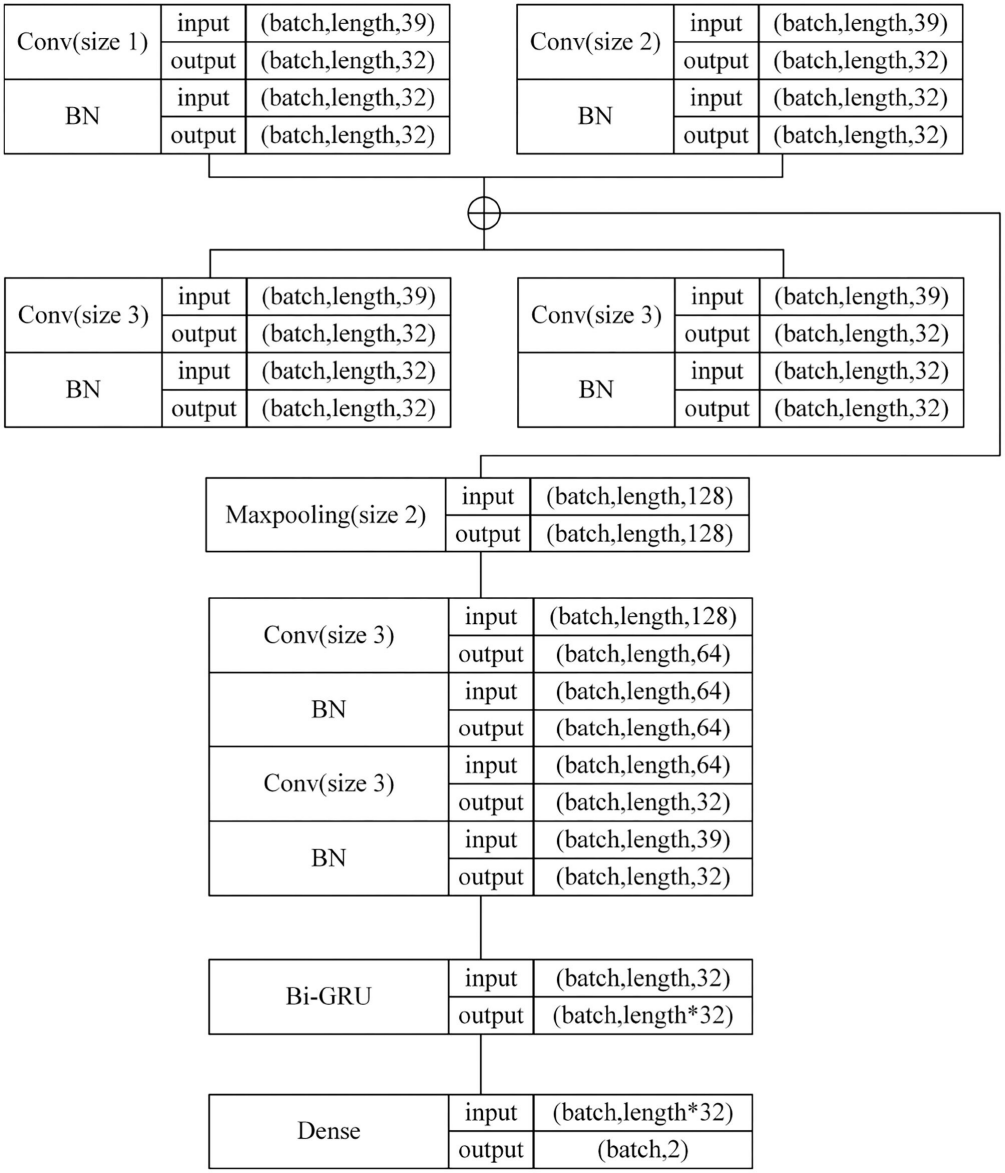
1D-CBBG network configuration details.

The One-Dimensional Convolutional filter Bank and Bi-GRU (Bidirectional GRU) model (in short 1D-CBBG) is improved on the basis of the CRNN, as shown in Figure 3. In the 1D multi-channel convolutional layer, the multi-scale frame-level features of speech are extracted by setting the convolution kernel of different sizes. In addition, the Bi-GRU is used to extract the high-dimensional features of speech context, which has a higher degree of fitting than the one-way recurrent network. Its network configuration details are shown in Figure 4. The input of the network are MFCC (Mel-Frequency Cepstral Coefficients) (Zaidan and Salam, 2016) features extracted from speech signals, and 39-dimensional MFCC features are extracted from each frame (each voice is represented as a feature matrix of length*39). Each speech is padded to the same length, and then goes through multi-channel convolution (convolution kernel size is 1, 2, 3, 4) and a BN (Batch Normalization) layer. The max pooling layer is used to increase the receptive field of the subsequent convolutional layer and reduce the number of parameters. After single-channel convolution, Bi-GRU is added to extract speech timing information. Finally, the output of GRU is flattened and input to the Softmax layer for classification probability calculation.

However, in daily conversation scenarios of depression patients, the performance of 1D-CBBG still has room for improvement. Firstly, the content of a dialogue is often fluid and closely related to the situation. Secondly, the scene is complex, and the background noise is large. Finally, the emotional information in daily speech is much weaker than in performance data sets. As a result, it is more difficult to recognize emotions in daily conversations. In order to more accurately identify the emotional information in speech, this section uses a three-dimensional speech feature to better represent the speech signal, and then improves the 1D-CBBG model.

### 3.1. Multi-Channel Convolutional Layer

**Figure 5:**
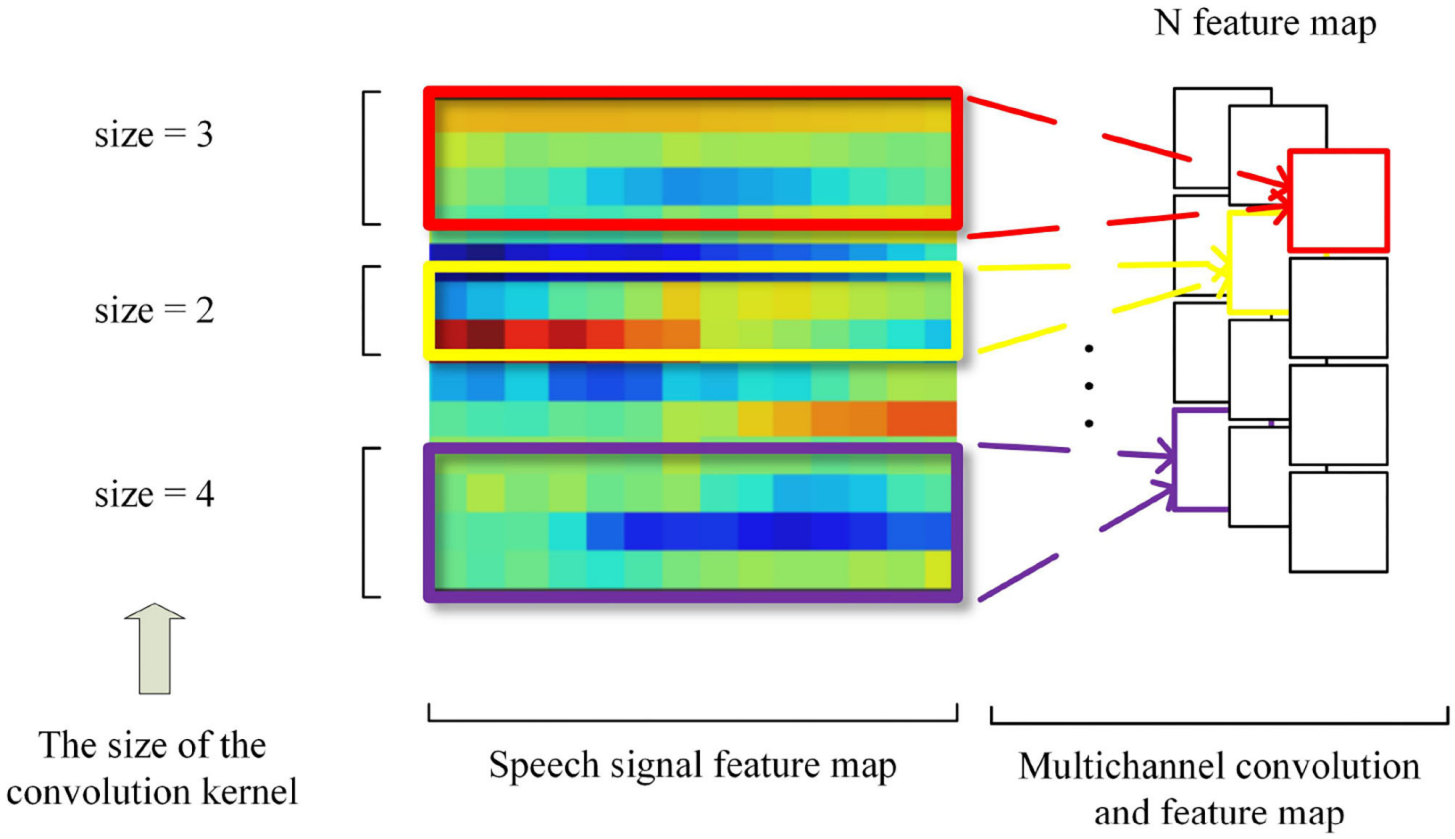
One-dimensional multichannel convolution.

Similar to the problem that the N-gram algorithm faces in text processing tasks, convolution usually has a fixed window size when processing speech features. But a speech signal has high coupling of local information, and the local region is not uniform. Therefore, it inspires us to apply one-dimensional multi-channel convolution to the emotion recognition task in speech frame-level local correlation feature extraction. For example, when the size of the convolution kernel is 2, 3, and 4, respectively, it is equivalent to extracting the local speech correlation of two, three, and four successive frames of the speech feature, respectively. The one-dimensional multi-channel convolution is shown in Figure 5.

Chan et al. found that two-dimensional convolution is superior to one-dimensional convolution under limited data, and time-domain convolution is as important as frequency-domain convolution (Chan and Lane, 2015). Inspired by the local perception mechanism in the CNN, in our subsequent work, the one-dimensional convolution in 1D-CBBG was replaced by the two-dimensional convolution to extract the local frame-level correlation of speech and carry out convolution calculation on the input of three-dimensional features.

### 3.2. Highway Networks

**Figure 6:**
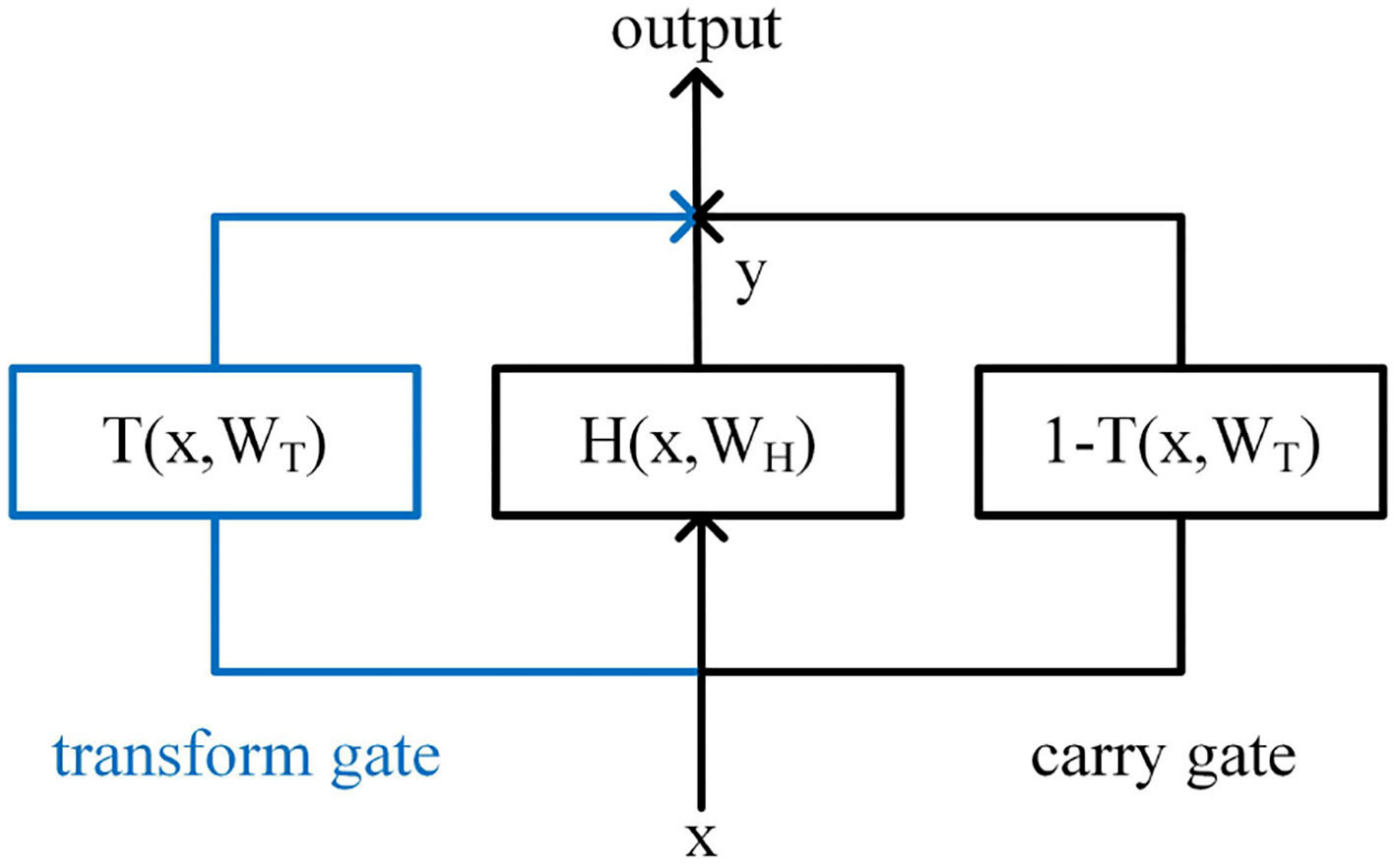
Highway Network structure.

Srivastava et al. proposed the Highway Network (Srivastava et al., 2015), which can train network parameters well when the networks are very deep. The structure of Highway is shown in Figure 6. Inspired by the gate mechanism of the LSTM, a transform gate *T* and a carry gate *C* are, respectively, set up. The calculation of *T* and *C* are shown in Equations (3) and (4).


(3) $$\begin{array}{l} T=\sigma_{1}\left(W_{T}x+b_{T}\right) \end{array}$$

(4) $$\begin{array}{l} C=\sigma_{2}\left(W_{C}x+b_{C}\right) \end{array}$$

Where, σ_1_ is usually the activation function Sigmoid, and σ_2_ is usually the activation function Relu.

The output of Highway Network is jointly determined by *T* and *C*, as shown in Equation (5). Simplify *C* to *C* = 1−*T*, and the final output is shown in Equation (6).


(5) $$\begin{array}{l} output=y⊙T+x⊙C \end{array}$$

(6) $$\begin{array}{l} output=y⊙T+x⊙\left(1-T\right) \end{array}$$

In the training process, through the parameter learning of *T*, the connection layer can be automatically assigned with the weight, so that the calculation of some connection layer can be skipped, and the output can be directly determined through *C* and the input *x*. While ensuring the network learning ability, the problem of gradient vanishing in the process of back propagation is effectively avoided.

### 3.3. Attention Mechanism

By simulating the human brain's characteristic that different types of information have different concerns, Mnih et al. proposed the attention mechanism (Mnih et al., 2014) and applied it to image recognition, which determined its contribution to the classification task by calculating the weight of different features. Bahdanau introduced the attention mechanism into the machine translation task in the field of NLP (Natural Language Processing) (Bahdanau et al., 2014), proving that it not only can better serve the image task, but also plays an important role in NLP. With the 2017 paper by Google's machine translation team, the attention mechanism has been widely applied in various fields and become a research focus of neural networks. Since the process of speech recognition is similar to a machine translation task in that it can be viewed as converting a given sequence into another sequence, the attention mechanism is also appropriate (Chorowski et al., 2015). In addition, in speech emotion recognition, it is used to increase the proportion of valid frames and reduce the interference of invalid frames, so as to improve the recognition rate of emotion. Huang et al. (Huang and Narayanan, 2017) proposed a Deep Convolutional Recurrent Neural Network (DCRNN) for speech emotion recognition, which uses the convolutional attention mechanism to learn the discourse structures related to tasks, thus reducing the probability of misclassification. Mirsamadi et al. (Mirsamadi et al., 2017) added the local attention mechanism on the basis of the RNN, so that the extracted global features can better pay attention to and reflect the emotional information in local features.

In the depression detection scenario, the attention mechanism also played an excellent role. Lu et al. (2021) proposed an emotion-based attention network that can capture high-level emotional semantic information and effectively improve depression detection tasks. A dynamic fusion strategy is proposed to integrate positive and negative emotional information. Zhang et al. (2020) combined demographic factors in EEG modeling and depression detection, integrating gender and age factors into a 1D CNN through attention mechanisms to explore the complex correlation between EEG signals and demographic factors, and ultimately to generate more effective high-level representations for the detection of depression. Based on the premise that different bands of the voice spectrum contribute unevenly to the detection of depression, Niu et al. (2021) proposed a time-frequency attention (TFA) component that highlights those distinct timestamps, bands, and channels that make the prediction of individual depression more effective than before.

Inspired by the previous work, the attention mechanism was also used in speech-based depression detection. Due to the different importance of the features of each frame in speech for emotion recognition, more attention should be paid to the frames with full emotion, on the contrary, the frames with poor emotional information should be reasonably ignored. Therefore, we introduced an attentional mechanism in a Bi-GRU for the detection of depression.

### 3.4. 3D Feature Extraction

**Figure 7:**
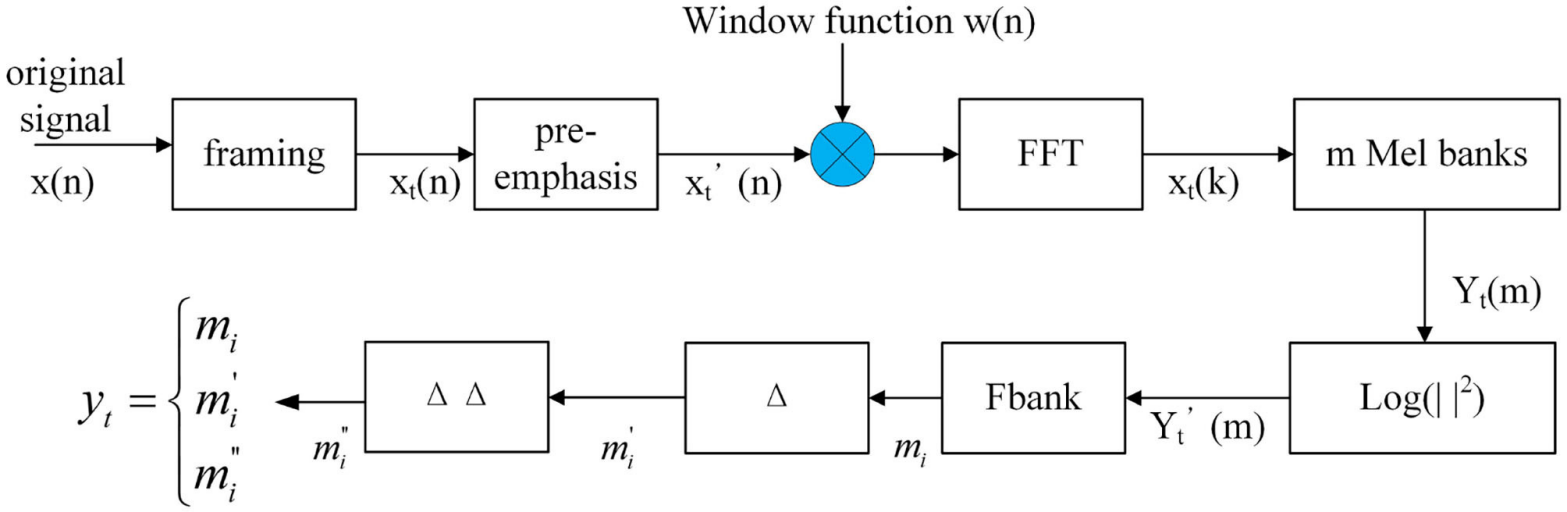
3D Fbank feature extraction.

Inspired by the dynamic difference of MFCC (including first-order and second-order difference) (Schuller et al., 2010), and in order to obtain more effective information from a speech signal, a three-dimensional feature extraction method is used. Firstly, speech is divided into frames and a 40-dimensional Fbank (Filter bank) (Swietojanski et al., 2014) feature is extracted as the low-dimensional feature. Then, the first-order difference $m_{i}^{\prime }$ and second-order difference $m_{i}^{\prime \prime }$ are calculated for each frame of speech *m*_*i*_, as shown in Equations (7) and (8), which can obtain the dynamic features and better represent the temporal correlation of speech. Finally, for each frame the speech will get the 40-by-3 feature. The feature extraction process is shown in Figure 7.


(7) $$m_{i}^{\prime }=\frac{\sum_{n=1}^{N}n(m_{i+n}+m_{i-n})}{2\sum_{n=1}^{N}n^{2}}$$

(8) $$m_{i}^{\prime \prime }=\frac{\sum_{n=1}^{N}n(m_{i+n}{}^{\prime }+m_{i-n}{}^{\prime })}{2\sum_{n=1}^{N}n^{2}}$$

### 3.5. Network Model

**Figure 8:**
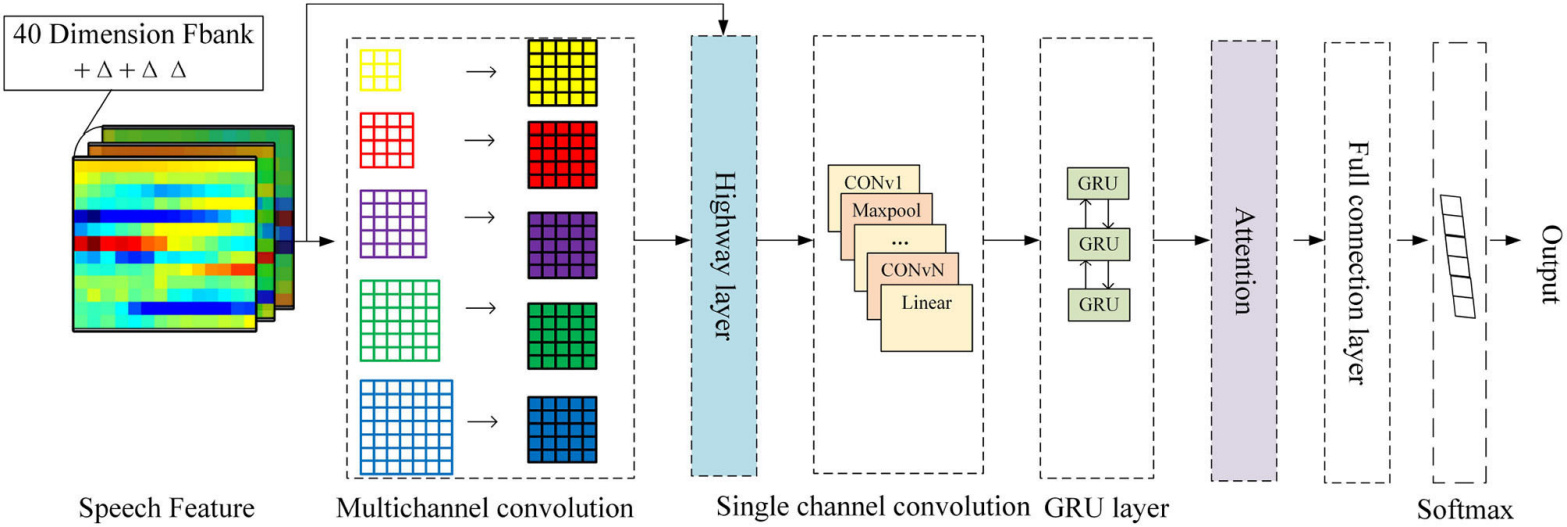
3D-CBHGA model.

In order to make full use of the information in speech signals, a 3D-CBHGA model is proposed in this section, and its structure is shown in Figure 8. The input of the model is a 3D feature extracted from the original speech, and the size is Paddinglength*40*3, where *Paddinglength* is the average length of the entire statement. Due to the variable length of the dialogue, the mode of all statements is taken and the average length is calculated. Then the long statement is divided into several pieces according to the average length and the short sentences are taken out. Finally, all the feature lengths are added to the same size *Paddinglength*. The multi-channel convolution layer is conducive to the feature extraction and learning of the N-gram pattern for speech signals. This layer is also used in 3D-CBHGA to carry out convolution calculation of the input 3D features. In the convolution process, the output is kept the same size as the input.

In order to better learn the high-dimensional features of speech signals, the Highway layer was introduced. *H* and *T* of the Highway are both composed of a fully connected network. By learning the proportion between the multi-channel convolution and the straight route, the speech features can be better represented. In this paper, the activation function of *H* is Relu, as shown in Equation (9).


(9) $$\begin{array}{l} f\left(x\right)=max\left(0,x\right) \end{array}$$

It attaches importance to the forward signal and ignores the reverse signal, which is similar to the response of human neurons to the signal. The activation function of *T* is Sigmoid, as shown in Equation (10).


(10) $$\begin{array}{l} f\left(x\right)=\frac{1}{1+e^{-x}} \end{array}$$

Its independent variable ranges from minus infinity to infinity, while the corresponding dependent variable is compressed to a range of 0 to 1. Therefore, *T* can be used as a gate structure to control the proportion of output.

**Figure 9:**
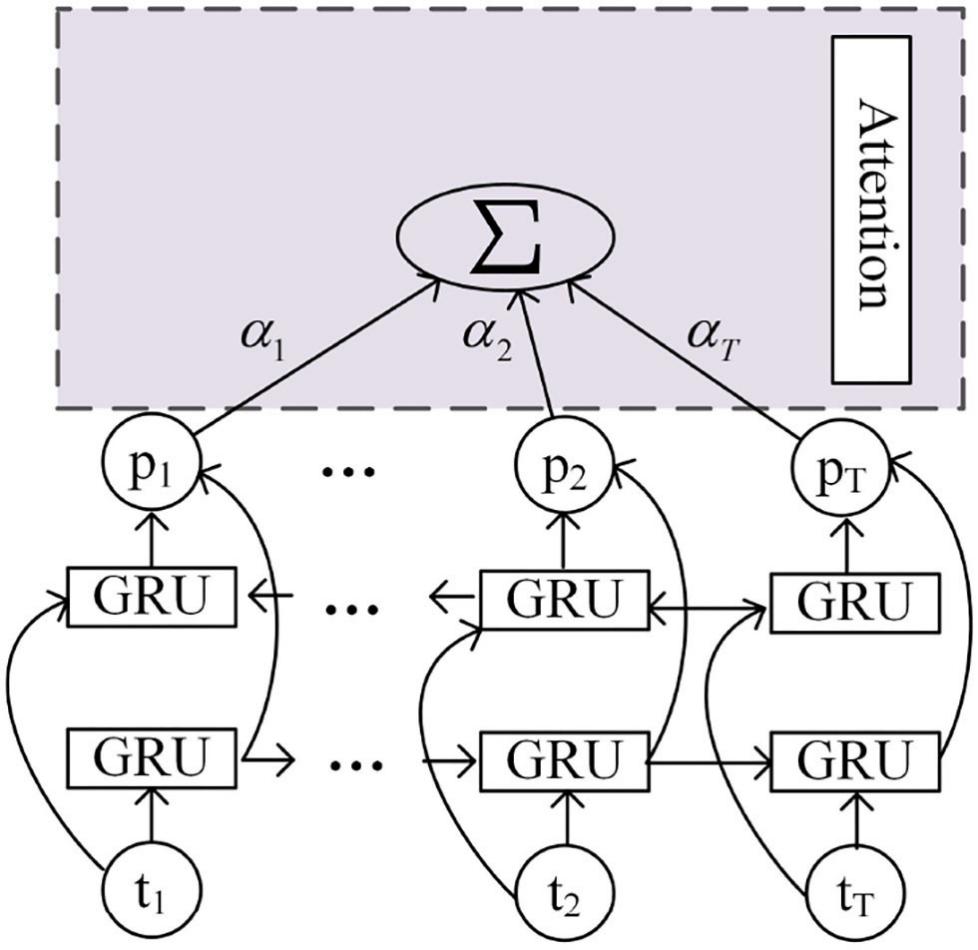
Bi-GRU based on attention.

After the Highway layer are the convolution layer and the Bi-GRU layer. Based on the different importance of each frame feature to speech emotion recognition, the attention mechanism is combined with the Bi-GRU structure, which is shown in Figure 9.

First, the weight corresponding to the output of GRU in each step is calculated through Equation (11), and then all the outputs are added with weights, as shown in Equation (12). The output of attention is obtained and passed into the dense layer for classification.


(11) $$\begin{array}{l} \alpha_{t}=\frac{exp\left(W\cdot p_{t}\right)}{\underset{T}{\sum }exp\left(W\cdot p_{t}\right)} \end{array}$$

(12) $$\begin{array}{l} output=\underset{T}{\sum }\alpha_{t}p_{t} \end{array}$$

Where *p*_*t*_ represents the output of the time step *t*, and α_*t*_ represents the weight of *p*_*t*_.

**Figure 10:**
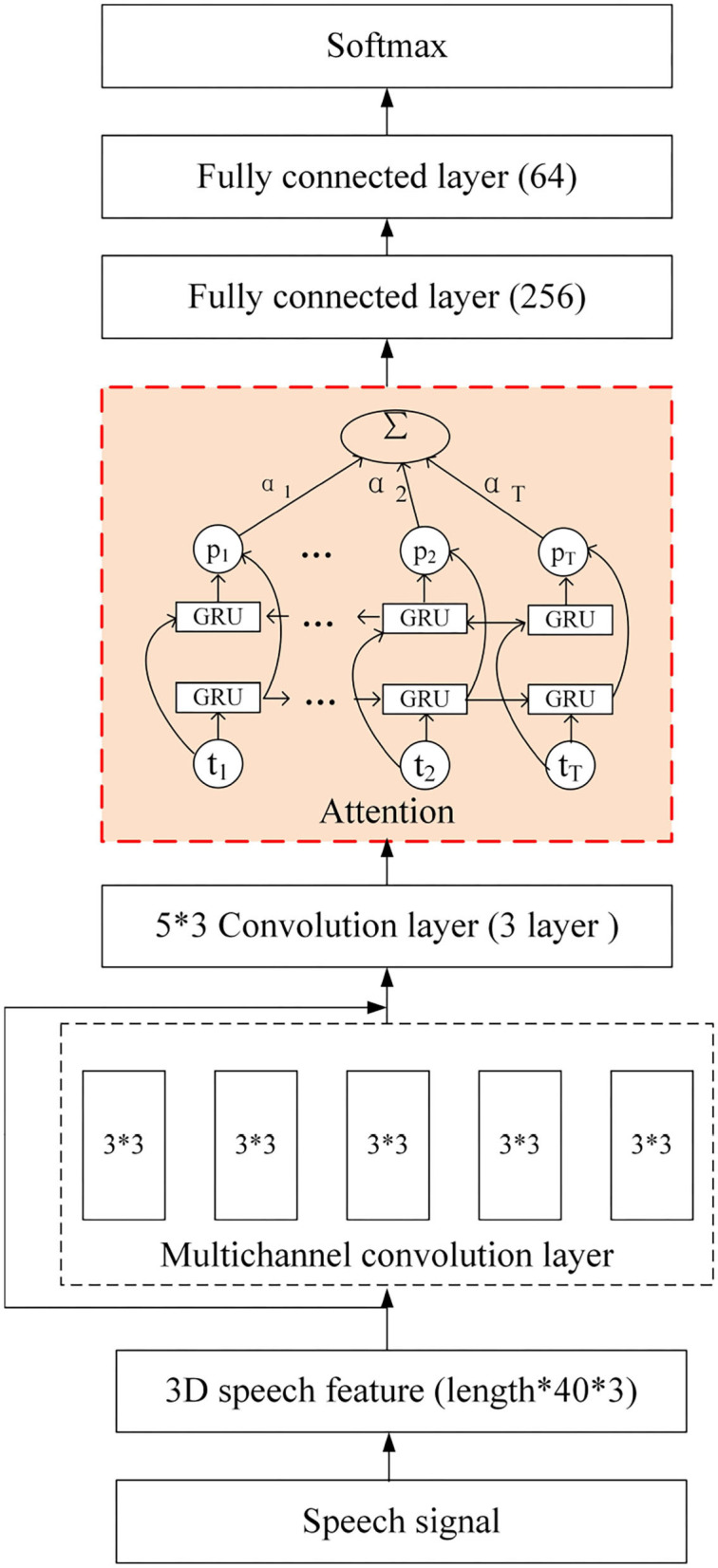
3D-CBHGA network configuration details.

The detailed network configuration of 3D-CBHGA is shown in Figure 10.

## 4. Experimental Results and Analysis

The 3D-CBHGA model proposed in this paper was applied to depression detection with other classical models, and the results of performance comparison were analyzed.

### 4.1. Experiment Settings

The experimental data set was the DAIC-WOZ English dataset, which is mainly used to analyze psychological disorders such as anxiety and depression, including speech, video, and questionnaire data. In this paper, only the speech signal is analyzed and tested. Participants were interviewed by Ellie, a virtual visitor, for a total of 189 interactions. The audio files and facial features of the participants in each session were recorded. The duration of each interaction ranged from 7 to 33 min (an average of 16 min).

Before the conversation with Ellie, each respondent filled out a questionnaire about mental state (PHQ-8, Kroenke et al., 2008). The binary classification of depression and non-depression was carried out based on the PHQ-8 score, and can be used as the labels for the respondents. Some examples are shown in Table 1, including participant IDs, PHQ-8 binary labels (PHQ-8 scores ≥ 10), PHQ-8 scores, and some questions of the PHQ-8 questionnaire. The classification is 0 for non-depression, and 1 for depression.

**Table 1:** DAIC-WOZ sample examples.

**Participant ID**	**Classification**	**PHQ-8 score**	**Gender**	**No interest**	**Depressed**	**Sleep**	**Tired**	**Appetite**	**Failure**	**Concentrating**	**Moving**
304	0	6	0	0	1	1	2	2	0	0	0
317	0	8	1	1	1	1	1	1	1	2	0
318	0	3	1	0	0	1	1	1	0	0	0
319	1	13	1	2	1	0	1	1	2	3	1
320	1	11	0	1	1	3	1	2	1	1	1
321	1	20	0	2	3	3	3	3	3	3	0

The 189 interactions were officially divided into 107 training sets, 35 verification sets, and 47 test sets (Gong and Poellabauer, 2017). However, in the test sets, only the gender information of the interviewees is given, and the label of whether they are depressed is not provided. Therefore, the training set and verification set (as test set) are only used for experimental analysis. The sample distribution is shown in Table 2. Because the number of interviewees in the dataset is too small and the data amount of a single interviewee is too large, each speech segment is cut every 1.5 s for the experiment (the actual sample number after cutting is shown in the table parentheses).

**Table 2:** Dataset sample distribution.

**Datasets**	**Non-depressed individuals (classification 0)**	**Depressed individuals (classification 1)**
Training sets	77 (3,750)	30 (3,000)
Test sets	23 (1,250)	12 (1,000)

In each scenario, Ellie's full conversation with the interviewee was recorded. The speech was picked up by microphone and the sampling frequency was 16 kHZ. Although the speaker spoke clearly, there were large gaps in the dialogue, as well as Ellie's questions and some scene noise. Voice Activity Detection (VAD) technology (Ramirez et al., 2007) is commonly used to mute speech. It separates the silent fragments from the speech fragments by boundary detection, and removes the long silent period in the original speech signal. We used the PyAudioAnalysis toolkit to segment the voice of the interviewees, the voice of Ellie, and the silent part in a voice sample file (window size and step in seconds are set to 0.020), and combined only the voice of the interviewees, so as to realize speech preprocessing. Finally, two complete data sets were obtained by DAIC-WOZ: DAIC-ori, the original complete dialogue data set, and DAIC-mute-removed, the data set with the mute segment removed.

Since the experiment was a binary classification task and the DAIC-WOZ sample was unbalanced, accuracy (in short *acc*), precision (in short *pre*), *error*, and *F*1 values of depression recognition are selected as evaluation indicators.

The calculation process of *acc* is shown in Equation (13). The higher the value is, the more samples the model detects correctly, and the better the effect will be.


(13) $$\begin{array}{l} acc=\frac{CS}{TS}\times 100 \end{array}$$

Where *CS* stands for the number of correct detection samples and *TS* stands for the total number of samples.

The calculation process of *pre* is shown in Equation (14). This value represents the correct number in a sample detected as depression. The higher the value, the higher the accuracy of the model in predicting depression.


(14) $$\begin{array}{l} pre=\frac{CSD}{TSD}\times 100 \end{array}$$

Where *CSD* stands for the number of samples correctly detected as depression and *TSD* stands for the total number of samples detected as depression.

The calculation process of *error* is shown in Equation (15). The value represents the proportion of the sample that misjudged the outcome as depressed to that confirmed as depressed. The lower the value, the lower the misjudgment rate of depression, and the better the model effect. *pre* and *error* are complementary indicators.


(15) $$\begin{array}{l} error=\frac{MSD}{TSD}\times 100 \end{array}$$

Where *MSD* stands for the number of samples misjudged as depression and *TSD* stands for the total number of samples detected as depression.

The experiments were carried out using Ubuntu 18.04, Python 3.6.9, and Tensorflow 1.13.1 with Intel(R) Core(TM) i7-9700K CPU and 32G Memory.

The 3D-CBHGA architecture is implemented with the TensorFlow toolkit, and the parameters of the model were optimized by minimizing the cross-entropy objective function, with a batch of 60 samples, using the Adaptive Moment Estimation (Adam) optimizer. The initial learning rate is set to 0.00001 and the epoch is set to 5000.

### 4.2. Experimental Results and Analysis

Two groups of comparative experiments were conducted on DAIC-ori and DAIC-mute-removed, respectively, and four algorithms including 1D-CBBG, 3D-CBHGA, SVM, and RF were used to detect and identify depression. The following experiments were conducted for 10 independent replicates, and the results were averaged. It mainly focuses on two issues: (1) performance comparison between different algorithms; and (2) the influence of different data sets on the performance of the algorithm.

Table 3 shows the performance of four different models in the original uncut data set DAIC-ori. As can be seen from Table 3, in DAIC-ori, the accuracy of traditional classification algorithm SVM and RF is 62.86 and 68.57%, respectively, the accuracy of the 1D-CBBG model is 71.43%, and the accuracy of the 3D-CBHGA model is the highest (74.29%). It is proved that in uncut data sets, the 3D-CBHGA model proposed in this paper can improve the mapping ability from speech signals to depression-related features, and can better detect and analyze depression through speech.

**Table 3:** Performance of different models on DAIC-ori.

**Models**	***Acc (%)***	***Pre (%)***	***Error (%)***	***F1***
SVM	62.86	41.67	58.33	0.455
RF	68.57	41.67	58.33	0.476
1D-CBBG	71.43	50.00	50.00	0.545
3D-CBHGA	**74.29**	**58.33**	**41.67**	**0.609**

*The values in bold are the data results produced by the model with the best performance in each indicator*.

Table 4 shows the performance of four different models in the DAIC-mute-removed data set after mute excision. According to Table 4, in DAIC-mute-removed, the 3D-CBHGA has the best performance in the four evaluation indexes. According to the comparison between Tables 3, 4, the performance of SVM, RF, 1D-CBBG, and 3D-CBHGA models on the DAIC-mute-removed is better than that in the original data set DAIC-ori, which proves that silent segments in speech conversations are interference items for depression recognition tasks.

**Table 4:** Performance of different models on DAIC-mute-removed.

**Models**	***Acc***	***Pre***	***Error***	***F1***
SVM	68.57	45.45	54.55	0.476
RF	71.43	54.55	45.45	0.522
1D-CBBG	74.29	58.33	41.67	0.609
3D-CBHGA	**77.14**	**63.64**	**36.36**	**0.636**

*The values in bold are the data results produced by the model with the best performance in each indicator*.

**Figure 11:**
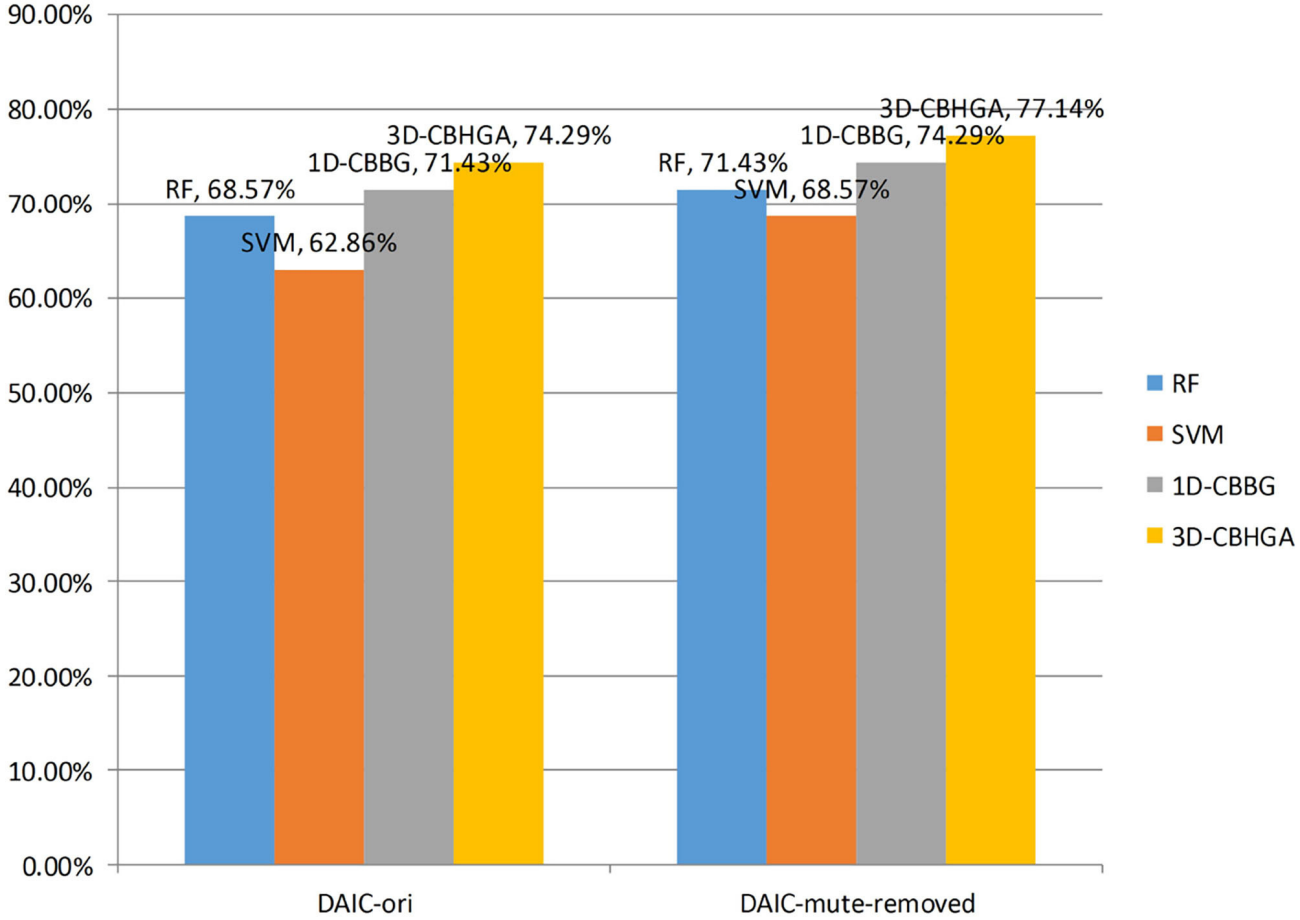
Accuracy of different models.

In order to better compare the algorithms, the accuracy of the four algorithms in the two data sets is drawn as shown in Figure 11. As can be seen from Figure 11, the accuracy of the 3D-CBHGA model is the highest among the four models, indicating that it has a strong ability to detect depression in speech. Due to the imbalance between the two data sets, the number of non-depressed samples is about twice that of depressed samples, so it is not accurate to judge the model only from the accuracy. Therefore, the *F*1 values of different models in the depression category are compared.

**Figure 12:**
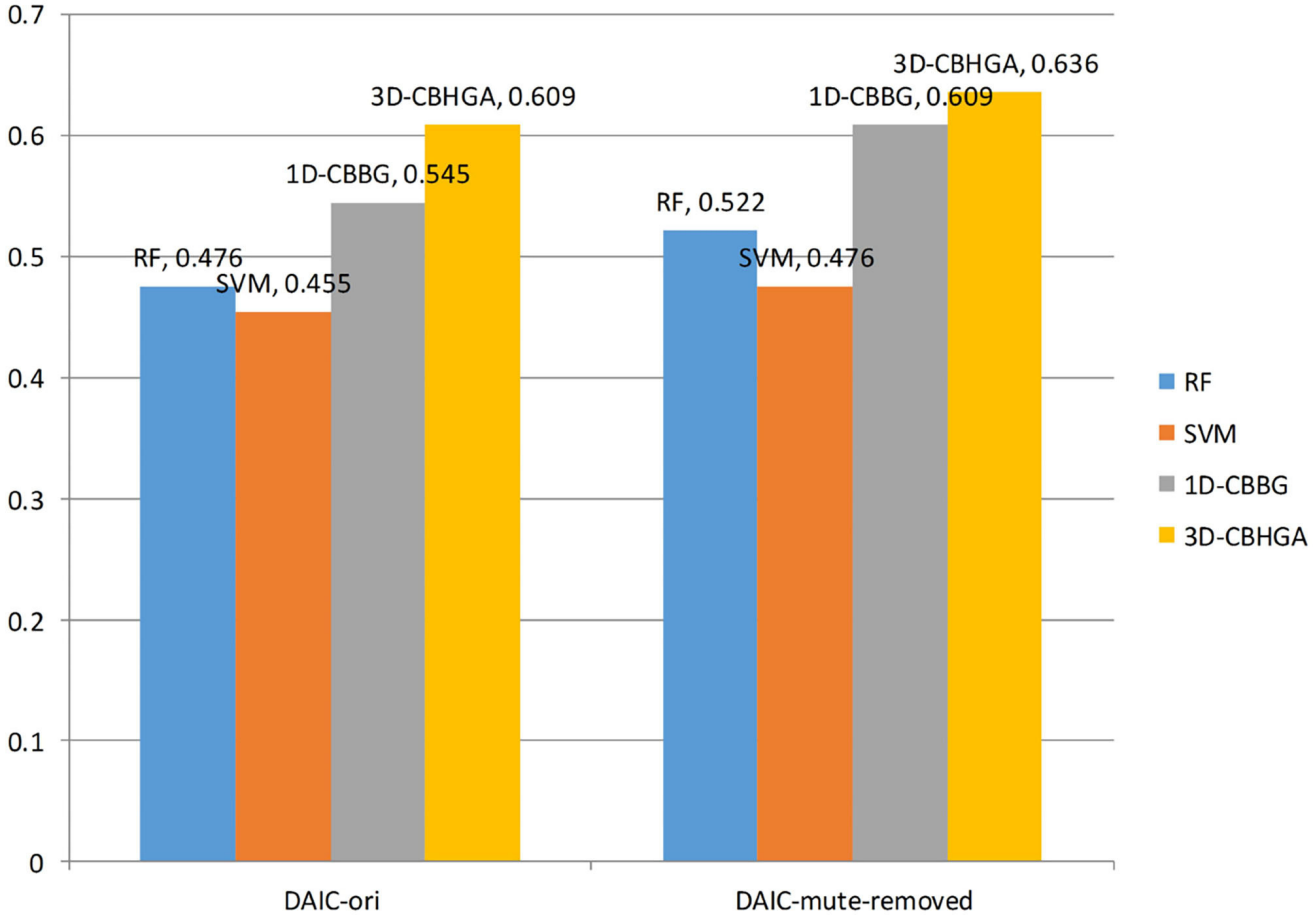
F1 values of different models.

The *F*1 values of the four algorithms in the two data sets are plotted as shown in Figure 12. As can be seen from Figure 12, the *F*1 value of the 3D-CBHGA model is the highest in the two data sets. In the DAIC-ori data set, the *F*1 value of 3D-CBHGA was 0.609, and that of SVM was 0.455. The 3D-CBHGA exceeded the SVM in F1 index by 33.8%. It proves that the model proposed in this paper can play a role in depression detection. At the same time, through the analysis of *F*1 value, it can be seen that in the dichotomy of depression and non-depression, the recognition performance of the four models for the depression category is low, and that of the non-depression category is high. The reason may be the imbalance between the non-depressed sample and the depressed sample in the data set.

## 5. Conclusion and Discussion

Depression is a severe mental health disorder with high societal costs. Speech signal characteristics can be one of the objective indicators for early recognition of depression. In order to solve the problem of small fluctuation of emotion in daily speech and limited ability of traditional feature extraction methods to represent speech signals, this paper proposes a feature enhancement method to extract three-dimensional features of speech. At the same time, a 3D speech emotion recognition model named 3D-CBHGA based on the combination of attention mechanism and Bi-GRU is proposed, and applied to a depression detection scenario. Finally, experiments show that it can improve the ability of depression detection and recognition. At the same time, it was proved that the model could improve the accuracy of depression detection by removing the blank fragment in speech.

In addition, this study only considered the differences between people with depression and healthy people, however, depression is often confused with other mental disorders such as bipolar disorder in clinical diagnosis, which can make diagnosis difficult. It is necessary to divide more detailed levels in future research. The future research work will focus on extracting other features of speech signals to better characterize them and learning among different languages to improve the reuse rate of the model.

## Data Availability Statement

The original contributions presented in the study are included in the article/supplementary material, further inquiries can be directed to the corresponding author/s.

## Author Contributions

XZ performed the experiment. HW and YL contributed significantly to the analysis and manuscript preparation, performed the data analyses, and wrote the manuscript. XT helped perform the analysis with constructive discussions. All authors agree to be accountable for the content of the work.

## Funding

This work is supported by the National Natural Science Foundation of China (No. 61572074) and National Key Research and Development Program of China (No. 2020YFB1712104).

## Conflict of Interest

The authors declare that the research was conducted in the absence of any commercial or financial relationships that could be construed as a potential conflict of interest.

## Publisher's Note

All claims expressed in this article are solely those of the authors and do not necessarily represent those of their affiliated organizations, or those of the publisher, the editors and the reviewers. Any product that may be evaluated in this article, or claim that may be made by its manufacturer, is not guaranteed or endorsed by the publisher.
